# Promising Cytokine Adjuvants for Enhancing Tuberculosis Vaccine Immunity

**DOI:** 10.3390/vaccines12050477

**Published:** 2024-04-29

**Authors:** Xuezhi Cao, Yang-Xin Fu, Hua Peng

**Affiliations:** 1 State Key Laboratory of Respiratory Disease, National Clinical Research Center for Respiratory Disease, Guangzhou Institute of Respiratory Health, The First Affiliated Hospital of Guangzhou Medical University, Guangzhou 510182, China; cao_xuezhi@gzlab.ac.cn; 2 Guangzhou National Laboratory, Bio-Island, Guangzhou 510005, China; 3 Department of Basic Medical Sciences, School of Medicine, Tsinghua University, Beijing 100084, China

**Keywords:** cytokine, adjuvant, vaccine, immunity, infection, mycobacterium, tuberculosis

## Abstract

Tuberculosis, caused by *Mycobacterium tuberculosis* (*M. tuberculosis*), remains a formidable global health challenge, affecting a substantial portion of the world’s population. The current tuberculosis vaccine, bacille Calmette–Guérin (BCG), offers limited protection against pulmonary tuberculosis in adults, underscoring the critical need for innovative vaccination strategies. Cytokines are pivotal in modulating immune responses and have been explored as potential adjuvants to enhance vaccine efficacy. The strategic inclusion of cytokines as adjuvants in tuberculosis vaccines holds significant promise for augmenting vaccine-induced immune responses and strengthening protection against *M. tuberculosis*. This review delves into promising cytokines, such as Type I interferons (IFNs), Type II IFN, interleukins such as IL-2, IL-7, IL-15, IL-12, and IL-21, alongside the use of a granulocyte–macrophage colony-stimulating factor (GM-CSF) as an adjuvant, which has shown effectiveness in boosting immune responses and enhancing vaccine efficacy in tuberculosis models.

## 1. Introduction

*M. tuberculosis*, a respiratory pathogen, is estimated to have infected nearly a quarter of the global population, encompassing between two billion and three billion individuals who are potentially at risk of developing tuberculosis [1,2]. While predominantly a pulmonary pathogen, *M. tuberculosis* can cause disease systemically. Tuberculosis manifests along a dynamic spectrum, ranging from asymptomatic infection to potentially fatal illness [3]. Tuberculosis ranks among the top ten leading causes of mortality worldwide and stands as the primary cause of death attributed to infection by a single pathogen [4]. The countries bearing the most tremendous burden of tuberculosis are India, Indonesia, China, Nigeria, Pakistan, and South Africa, collectively representing around 60% of the worldwide tuberculosis incidence [5]. To achieve the End Tuberculosis Strategy, the World Health Organization’s ambitious goal of eradicating the tuberculosis epidemic by 2035, the targets are set at a 95% reduction in tuberculosis-related deaths and a 90% reduction in tuberculosis incidence [6]. While tuberculosis can be effectively cured with drug therapy, the rising prevalence of drug-resistant strains diminishes the efficacy of this approach. Therefore, achieving control over tuberculosis necessitates a comprehensive strategy involving improved medications, diagnostics, and vaccines [7,8].

The only licensed vaccine for tuberculosis prevention is BCG, initially administered in Paris in 1921, marking over a century of continuous utilization [9]. BCG offers durable and potent protection against miliary and meningeal tuberculosis in children. However, its efficacy in preventing pulmonary tuberculosis, notably in adults across various clinical trials, has been suboptimal, contributing to its ineffectiveness in stemming the global epidemic [9,10]. In a recent phase II trial conducted in a high-risk setting, BCG revaccination demonstrated a 45.4% efficacy in reducing a sustained QuantiFERON-TB Gold In-tube assay (QFT) conversion rate among adolescents who had received a neonatal BCG vaccination. This outcome was used to evaluate the protective efficiency against reactivation from a latent TB infection [11]. GSK’s subunit tuberculosis vaccine M72/AS01E has recently concluded phase II clinical trials [12]. This M72/AS01E vaccine comprises the M72 recombinant fusion proteins derived from Mtb32A and Mtb39A, adjuvanted with AS01E, composed of monophosphoryl lipid A (MPL) and the saponin QS-21. The primary analysis, conducted two years after the second vaccination, revealed a 49.7% reduction in active TB cases among individuals who received the M72/AS01E vaccine compared to those who received the placebo [13]. These promising clinical findings from the BCG revaccination and M72/AS01E trials kindle hopes for the development of highly effective tuberculosis vaccines. Furthermore, they also underscore the potential for significant advancements in tuberculosis vaccine efficacy in future investigations.

Identifying immune signatures as immunological correlates of protection (CoP) is pivotal for streamlining vaccine development and comparison [14]. Considering the critical role of IFN-γ in tuberculosis immunity, it was hypothesized that T-cell secretion of IFN-γ might serve as a CoP. However, while IFN-γ is indeed indispensable for tuberculosis immunity, its sole presence does not suffice to confer protection [14]. Antigen-specific Th1/17-type responses have conferred protection across various non-human primate (NHP) studies, including those induced by bronchoscope-delivered BCG [15,16]. Despite initial underestimation, antibody responses have emerged as significant correlates of protection. Recent clinical data have underscored a connection between antibody titers and reduced susceptibility to infection post-BCG vaccination [17]. Additionally, trained innate immunity has been acknowledged as a CoP against *M. tuberculosis* infection. BCG vaccination-induced trained innate immunity potentially enhances the early clearance of *M. tuberculosis* [18,19]. T cells warrant particular attention as they play a crucial role in preventing a primary disease upon initial *M. tuberculosis* infection, as well as in the development of post-primary tuberculosis once a latent infection has been established [20]. The long-lived MTB-specific memory T (TM) cells have the potency to mount a swift and potent immune response to the pathogen re-exposure, thereby substantiating the efficacy of the vaccination [20]. These TM cells encompass various subsets, including central memory (TCM), effector memory (TEM), tissue-resident memory (TRM), and stem-cell-like memory (TSCM) T cells [21]. One promising strategy for effectively controlling tuberculosis through vaccination involves augmenting the generation of a larger pool of durable memory T cells. 

Cytokines act as crucial immune system regulators, playing vital roles in maintaining a physiological balance and influencing pathological conditions [22]. Various cytokines have proven effective as immunological adjuvants in diverse model systems, enhancing the protective efficacy of vaccines against viral, bacterial, and parasitic infections. The strategic use of cytokines presents an opportunity to selectively boost specific immune parameters, thereby enhancing protective outcomes and mitigating potential adverse effects of vaccination [23]. Increasing evidence highlights the pivotal role of cytokines in the differentiation of memory T cells and suggests their potential contribution to the heightened basal turnover rate observed in these cells [24,25]. In this review, we discuss recent investigations of various cytokines as adjuvants in tuberculosis vaccines, exploring their impact on vaccine-triggered T-cell responses and their roles in conferring protection against tuberculosis (Table 1).

## 2. Type I IFNs

The Type I IFN family stands out as a multifaceted cytokine group encompassing 13 partially homologous IFNα subtypes in humans (14 in mice), alongside a singular IFNβ and several ambiguously characterized single gene products, namely IFN-ɛ, IFN-τ, IFN-κ, IFN-ω, IFN-δ, and IFN-ζ [55,56]. Type I IFNs exert a wide range of effects on both innate and adaptive immune cells in response to viral, bacterial, parasitic, and fungal infections, either directly or indirectly, by triggering the expression of other downstream functional mediators [56]. Type I IFNs can stimulate the maturation of antigen-presenting cells (APCs), elevate the expression of costimulatory signals, and augment their capacity for antigen presentation or cross-presentation [57,58]. Studies conducted in murine models and human subjects have elucidated the involvement of IFN-α/β in directly modulating the differentiation of both CD4^+^ and CD8^+^ T cells upon an initial antigen encounter [59]. Moreover, IFN-α/β, alongside other innate cytokines, is recognized as a pivotal ‘third signal’ in determining the composition of the effector and memory T-cell reservoir [59]. Indeed, Type I IFNs have been established as valuable natural adjuvants for human vaccine formulations. Previous studies have reported a cytokine fusion protein-based COVID-19 vaccine platform. It is an interferon-armed RBD fusion protein incorporating a Pan DR-binding epitope (PADRE) T-helper epitope and Fc domain, named IPRF, suitable for intramuscular injections and intranasal vaccinations without additional adjuvants [60,61,62]. Based on this design, the human vaccine (V-01), developed by a subsidiary of Livzon Pharmaceutical Group Inc. (Zhuhai, China), underwent three clinical trial phases, demonstrating high neutralizing antibody responses and an excellent safety profile in both adult and elderly groups following an intramuscular vaccination [63]. Consequently, V-01 has received emergency use authorization in China as a booster vaccine.

A comparative analysis revealed that BCG is less effective in inducing dendritic cell (DC) maturation than *M. tuberculosis*, leading to the reduced expression of IFN-β and IL-12 in BCG-infected DCs compared to *M. tuberculosis*-stimulated cells. The supplementation of BCG-infected DCs with exogenous IFN-β, known for its immunomodulatory effects, enhanced the Th1-type response, promoting a mature phenotype and increased secretion of IL-12 [26]. Similarly, IFN-β-pretreated BCG-infected DCs exhibited markedly increased IL-12 secretion in comparison to both BCG-infected DCs and *M. tuberculosis*-infected cells [27]. In animal and clinical studies, combining intravesical BCG with IFN-α for superficial bladder cancer exhibits enhanced efficacy compared to either agent alone. IFN-α significantly boosts BCG-induced IFN-γ production in bladder cancer patients, with most patients experiencing a substantial increase. IFN-α also enhances BCG-induced IL-12 and TNF-α while reducing IL-10 levels. IFN-α enhances BCG’s immune response by promoting Th1-type cytokines and reducing Th2-type cytokines [28]. In another study, consecutive boosts of IFN-α in BCG-vaccinated mice protected against *M. lepraemurium* infection [29]. Of particular significance, intramuscular co-administration of IFN-α with the BCG vaccine was demonstrated to enhance specific anti-mycobacterial Th1-type cytokine production in both in vitro and in vivo settings, leading to a reduction in the bacterial burden after the *M. tuberculosis* challenge. This reduction amounted to 0.3 logs in the lungs and a noteworthy 0.9 log decrease in bacterial load in the spleen compared to mice vaccinated solely with BCG [30].

The precise roles of Type I IFNs in both the pathogenesis and control of mycobacterial infections are still controversial and contingent upon the experimental conditions. One clinical isolate of *M. tuberculosis*, HN878, was found to be exceptionally virulent, leading to early death in immune-competent mice. HN878 infection elevated the levels of Type I IFNs, further suppressing Th1-type immunity [64]. In vitro, monocytes demonstrated effective control over the growth of *M. bovis* BCG. Uncontrolled mycobacterial growth was observed when monocytes were exposed to Type I IFNs, suggesting that Type I IFNs may develop a favorable intracellular environment to promote mycobacterial growth [65]. On the contrary, administering aerosolized IFN-α to patients undergoing antimicrobial therapy resulted in a swifter reduction in the bacilli counts detected in sputum and an amelioration of pulmonary tuberculosis [66,67]. Recent studies have established the role of Type I IFNs as innate immune enhancers for commercial vaccines against SARS-CoV-2 [60,62,63,68,69], offering promise for the potential utilization of Type I IFNs as an adjuvant in combating other pathogens. Type I IFNs improve dendritic cell functionality post-BCG infection, potentially acting as a valuable adjuvant to boost BCG immunogenicity. Moreover, Type I IFNs show promise in regulating the T-helper cell-mediated immune response, thereby enhancing BCG-induced immunity against *M. tuberculosis* infections. In conclusion, owing to their immunomodulatory properties and extensive clinical track record, Type I IFNs stand out as promising candidates for adjuvant use in vaccination against pathogenic mycobacterial infections.

## 3. Type II IFN

The Type II IFN family comprises a singular gene product, IFN-γ, primarily synthesized by T cells and natural-killer (NK) cells. IFN-γ exhibits its biological effects on diverse cell types expressing the IFN-γ receptor (IFNγR) [56,70]. Biologically, IFN-γ is a pleiotropic cytokine with antiviral, antitumor, and immunomodulatory properties, thereby serving a crucial function in orchestrating both innate and adaptive immune responses [71,72]. By acting on APCs, IFN-γ enhances the expression of costimulatory molecules and cytokines essential for activating T cells [73]. Precise levels of IFN-γ appear to be essential for the viability and functionality of effector memory CD4^+^ T cells [74]. IFN-γ facilitates the proliferation of low-avidity T cells, enabling them to surpass the competitive edge of high-avidity T cells while also enhancing the incorporation of high-avidity T cells into the memory reservoir. This process ultimately lowers the average avidity of the initial response and elevates that of the memory response [75]. The therapeutic potential of IFN-γ against tuberculosis and multidrug-resistant tuberculosis (MDR-TB) has been extensively investigated since the end of the last century. Multiple clinical trials have underscored the effectiveness of IFN-γ in treating tuberculosis [76]. While the clinical studies offer valuable insights, they represent only a fraction of the comprehensive evaluation required to ascertain the therapeutic capacity of IFN-γ in tuberculosis and related mycobacterial infections. More clinical trials are needed to refine our understanding and delineate the precise therapeutic potential of IFN-γ in this regard [77]. Alternatively, studies across diverse animal models have reported the potential utility of various forms of IFN-γ as adjuvants for vaccines [78].

A multivalent vaccine containing six recombinant antigens (Ag85B, Rv0934, ESAT-6, CFP21, Mtb8.4, and Rv2031c) from *M. tuberculosis* was examined in mice, in conjunction with a Ribi (monophosphoryl lipid A-trehalose dicorynomycolate) adjuvant [79] and IFN-γ, leading to a marked reduction in colony-forming unit (CFU) counts upon exposure to a virulent *M. tuberculosis* strain, mirroring the protective efficacy of the BCG vaccine [31]. Moreover, splenocyte proliferation, IFN-γ secretion, and nitric oxide (NO) production were significantly elevated in splenocytes derived from mice immunized with Ribi + 6Ag + IFN-γ, in contrast to those from mice immunized with Ribi + 6Ag [31]. Another approach evaluated the protective effectiveness of a novel recombinant BCG strain (rBCG-AEI) expressing a fusion protein comprising antigens Ag85B, ESAT-6, and IFN-γ against *M. tuberculosis* H37Rv in murine models. The rBCG-AEI elicited heightened specific antibody titers and significantly bolstered cellular immune responses when contrasted with BCG, rBCG-A (expressing Ag85B), and rBCG-AE (expressing Ag85B-ESAT-6) [32]. Protective assays illustrated that rBCG-AEI conferred comparable or superior protection against *M. tuberculosis* infection regarding organ bacterial burdens, lung-histopathological changes, and weight loss, underscoring its potential as a promising candidate warranting further exploration [32]. These results confirm the establishment of a vigorous cellular immune response bolstered by IFN-γ in vaccinated mice, correlating with heightened resilience against *M. tuberculosis*. An investigation evaluated the effects of a recombinant BCG expressing IFN-γ (BCG-IFN) on inflammation and tissue fibrosis. Notably, intravenous administration of BCG-IFN resulted in decreased organ weight and bacterial load by day 21 in comparison to control BCG-plasmid administration. Furthermore, a reduction in inducible nitric oxide synthase (iNOS) mRNA, iNOS^+^ cells, granulomas, and liver hydroxyproline content with BCG-IFN suggested improved bacterial clearance and diminished tissue pathology at mycobacterial infection sites [33]. These findings illustrate that the localized expression of IFN gamma by the recombinant BCG enhances bacterial clearance, leading to a concomitant reduction in tissue pathology. This effect mitigates the concern that heightened immunoreactivity could exacerbate vaccination-related tissue damage.

The assessment of the IFN-γ response to *M. tuberculosis* infections has been utilized in both research and clinical settings to establish and evaluate new strategies for preventing, diagnosing, and treating such infections [80]. The production of IFN-γ serves as a functional marker for murine T cells that impart adaptive immunity against *M. tuberculosis* [81]. Specifically, IFN-γ plays a pivotal role in developing protective immunity against *M. tuberculosis* infections, serving as a crucial mediator in activating macrophages [82]. In conjunction with adjuvants and IFN-γ, the multivalent vaccine exhibited a notable decrease in bacterial counts and bolstered immune responses in murine subjects. Furthermore, the recombinant BCG strain expressing distinct antigens and IFN-γ displayed heightened efficacy in shielding against *M. tuberculosis* infections in murine models. Hence, IFN-γ is a promising candidate for incorporation as an adjuvant in tuberculosis vaccine formulations.

## 4. IL-2

IL-2 is a member of the IL-2 superfamily containing six kinds of cytokines, namely IL-2, IL-4, IL-7, IL-9, IL-15, and IL-21, all of which share a common γ chain [83]. The principal function of IL-2 is to initiate immune responses by promoting the proliferation and differentiation of effector T cells, memory T cells, and NK cells [84]. IL-2 serves as a regulator of IL-7Rα expression, thereby influencing CD4^+^ memory T-cell homeostasis in vivo [85]. Within CD8^+^ cells, IL-2 can promote cellular proliferation and drive differentiation towards memory and terminally differentiated lymphocytes [86]. IL-2 signals are critical for the formation of long-lived CD8^+^ T-cell memory [87]. Importantly, IL-2 has been reported as a promising adjuvant for various vaccines against viruses, bacteria, and tumors [88,89,90].

A genetically modified BCG strain was constructed to encode human IL-2 and the ESAT-6 antigen from *M. tuberculosis*. This engineered BCG variant induced robust Th1-type responses, marked by enhanced lymphoproliferation, IFN-γ secretion, and augmented cytotoxic T-lymphocyte functionality [34]. In another approach, a DNA vaccine encoding a fusion protein of *M. tuberculosis* heat shock protein 65 (Hsp65) with human IL-2-induced robust antigen-specific immune responses, including antibody generation, IFN-γ release, and activation of CD4^+^ and CD8^+^ T cells, following *M. tuberculosis* H37Rv infection. Mice vaccinated with the DNA construct displayed significantly reduced bacterial burdens in organs compared to the control cohort, albeit falling short of the efficacy observed with BCG. Histopathological analysis revealed attenuated pulmonary pathology in DNA-vaccinated mice akin to those in BCG-immunized counterparts, in stark contrast to the saline control group [35]. Likewise, mice immunized with HSP65-IL-2-DNA displayed a significant decrease in *M. tuberculosis* colony counts in the spleen and lungs following a challenge with virulent *M. tuberculosis* H37Rv. The HSP65-IL-2-DNA vaccine exhibited superior protective and therapeutic effects when contrasted with the HSP65-DNA vaccine, indicating that incorporating IL-2 in the DNA vaccine enhances its immunogenicity and effectiveness against *M. tuberculosis* by bolstering a Th1-type immune response [36]. Moreover, incorporating IL-2 into a recombinant BCG (rBCG-IL-2) vaccine elicited a Th1-type immune profile in both immunocompromised and IL-4 transgenic mice. Pre-vaccination administration of dexamethasone before rBCG-IL-2 or BCG inoculation resulted in distinct immune responses: rBCG vaccination triggered a robust Th1-type response characterized by IFN-gamma predominance, while BCG induced a Th2-type response with IgG1 dominance [37].

The stimulatory impact of IL-2 on effector T cells (Teff) and NK cells prompted investigations of high-dose IL-2 for cancer therapy, leading to the approval of recombinant human IL-2 (Aldesleukin) as the inaugural immunotherapy endorsed by the US Food and Drug Administration for managing metastatic renal cell carcinoma (RCC) in 1992 and metastatic melanoma in 1998 [91]. The considerable clinical experience with IL-2 has markedly enhanced its potential for advancing tuberculosis therapeutic agents and vaccine adjuvants. IL-2 has been substantiated as an efficacious therapeutic agent in managing MDR-TB [92]. In addition, continuous exposure to *M. tuberculosis* antigens resulted in T-cell dysfunction, which could be effectively reversed through supplementation with IL-2 [93]. Consequently, IL-2 emerges as a promising candidate for incorporation as an adjuvant in tuberculosis vaccine formulations.

## 5. IL-7 and IL-15

IL-7 and IL-15 are members of the IL-2 superfamily [94]. IL-7 is required for T-cell development and for maintaining and restoring homeostasis of mature T cells [95]. IL-15 exhibits a wide array of functions in the modulation of both adaptive and innate immune responses, mirroring the activities of IL-2 [96]. IL-7 and IL-15 exhibit a range of effects concerning T-cell survival, activation, clonal expansion, and the development and sustenance of memory cells. Specifically, IL-7 supports the survival of both naive and memory T cells, while IL-15 plays a crucial role in the homeostatic proliferation of memory CD8^+^ T cells and the preservation of a constant level of CD8^+^ T-cell memory [24]. The diverse biological functions of IL-7 underscore its significance as a crucial molecular adjuvant for enhancing vaccine efficacy [97]. The crucial role of IL-15 in fostering enduring immune memory and sustaining immune responses explains the significantly improved vaccine immunity when integrating IL-15 molecules into vaccine formulations [98,99].

In a murine model of *M. tuberculosis* infection, the simultaneous administration of nonlytic Fc-fused IL-7 DNA (IL-7-nFc) and Flt3-ligand-fused Mtb32 (F-Mtb32) DNA, alongside chemotherapy, significantly augmented Mtb32-specific T-cell responses, persisting for up to a year post the final immunization [38]. Concomitant delivery of IL-7-nFc and F-Mtb32 DNA also decreased *M. tuberculosis* reactivation following dexamethasone treatment, ameliorated lung pathology, and reduced pulmonary inflammation. The heightened protection observed with this combined approach was associated with increased Mtb32-specific IFN-γ-secreting CD4^+^ and CD8^+^ T-cell responses in the lungs and spleens, indicating the potential of IL-7-nFc as a promising adjunct for tuberculosis DNA vaccines in clinical applications [38]. 

Immunization with rBCG-Ag85B-IL-15 (a recombinant BCG expressing a fusion protein Ag85B-IL-15) elevates the levels of IFN-γ-producing CD8^+^ and CD4^+^ T cells, exceeding the response induced by a rBCG expressing Ag85B alone (rBCG-Ag85B), resulting in notable lung protection upon challenge with *M. tuberculosis*. The vaccination with rBCG-Ag85B-IL-15, known for its ability to trigger potent cell-mediated immunity, presents a promising avenue for an effective tuberculosis vaccine [39]. In another study, a modified vaccinia Ankara (MVA) construct, expressing five *M. tuberculosis* antigens and IL-15 (MVA/IL-15/5Mtb), exhibited enduring protective immunity lasting at least 16 months post-initial vaccination. Homologous prime/boost with MVA/IL-15/5Mtb demonstrated sustained protection on par with BCG immunization, characterized by heightened levels of crucial immune markers post-tuberculous challenge [40].

Co-administration of IL-7 and IL-15 with the BCG vaccine markedly amplifies the memory response of CD4^+^ and CD8^+^ T cells, resulting in increased T-cell proliferation, elevated production of Th1-type cytokines, and the expansion of multifunctional *M. tuberculosis*-specific memory T cells, in contrast to mice vaccinated solely with BCG. This enhancement significantly diminishes the mycobacterial load in the lungs, underscoring the promise of IL-7 and IL-15 supplementation in enhancing the effectiveness of the BCG vaccine [41]. Similarly, mice receiving tuberculosis subunit vaccines (LT70 [100] and MH [101]) in combination with recombinant adenovirus encoding fusion cytokines IL-7-Linker-IL-15 (rAd-IL-7-Linker-IL-15) regimen exhibited enhanced long-term immune responses and increased protective efficacy against the BCG challenge compared to the control cohorts. The potential of rAd-IL-7-Linker-IL-15 to augment the efficacy of tuberculosis subunit vaccines relies on its ability to strengthen central memory-like T cells, thereby providing enduring protection against *M. tuberculosis* [42].

IL-7 is crucial for providing the essential survival signal during the transition from effector to memory CD8^+^ T cells; however, the expression of the IL-7 receptor alone was not adequate [102]. Studies have revealed that the combined signaling of IL-7 and IL-15 synergistically fosters the development of memory T cells, highlighting the essential roles of both cytokines in the initiation and sustenance of memory CD4^+^ and CD8^+^ T cells [103,104]. Consequently, enhancing vaccines with IL-7 and IL-15 may offer a promising avenue to enhance the enduring maintenance of memory T cells over the long term. Particularly, the exogenous administration of IL-15 did not significantly affect the progression of *M. tuberculosis* infection, as demonstrated by the absence of significant variations in the bacterial burden or T-cell numbers between IL-15-treated mice and untreated controls [105]. In contrast, IL-15 secreted from the IL-15-expressing rBCG represents a viable approach for fostering T-cell immunologic memory triggered by BCG. Here, the sustained IL-15 release plays a crucial role in the maintenance of memory T cells, as opposed to the rapid decline in cytokine efficacy observed when cytokines are administered independently in the host [39]. In conclusion, the integration of IL-7 and IL-15 with existing tuberculosis vaccines has shown significant promise in enhancing immune responses and strengthening the defense against *M. tuberculosis* infections. It indicates a hopeful direction for advancing more robust and long-lasting tuberculosis prevention strategies.

## 6. IL-12

IL-12, a member of the IL-12 family, encompasses four cytokines: IL-12, IL-23, IL-27, and IL-35. IL-12 consists of two subunits, IL-12p35 and IL-12p40, necessitating their concurrent expression within a single cell to release the bioactive disulfide-linked IL-12p70 cytokine [106]. IL-12 is a pro-inflammatory cytokine that governs T-cell and natural-killer-cell responses, stimulates IFN-γ production, promotes the differentiation of Th1-type cells, and serves as a vital bridge between innate resistance and adaptive immunity [107]. In vivo studies have demonstrated that IL-12 enhances the expansion of CD8^+^ T cells and promotes the generation of memory cells during an immune response [108]. Given its immunostimulatory attributes, consistent interest persists in leveraging IL-12 as a vaccine adjuvant. IL-12 has been widely researched as an adjuvant for promoting protective immune responses, including antibody induction, cell-mediated immunity, and the enhancement of mucosal immunity [109,110,111].

Although BCG initially provided robust protection against early *M. tuberculosis* infection, its efficacy waned over time [43]. Researchers investigated the impact of IL-12 as an immune adjuvant in enhancing the effectiveness of BCG vaccination. Mice vaccinated solely with BCG displayed decreased bacterial loads when challenged with *M. tuberculosis*; however, more significant reductions were observed in those vaccinated with BCG in combination with IL-12. Enhanced IFN-γ production was detected in the spleen cells of mice that received BCG along with IL-12 [44]. Similarly, co-administration of an IL-12 containing DNA construct with BCG markedly elevated IFN-γ levels compared to BCG alone. The combined administration of IL-12 DNA vaccine constructs with BCG offered slightly improved protection in the early stages and significantly enhanced protection in later stages compared to BCG alone. This synergistic strategy elicited a more potent immune response and demonstrated superior effectiveness in combating progressive *M. tuberculosis* infection [43]. In another investigation, a plasmid encoding IL-12 markedly enhanced the protective efficacy of the DNA vaccine expressing Ag85B against the *M. tuberculosis* challenge by amplifying T-cell responses. IL-12 has emerged as a pivotal cytokine adjuvant for enhancing immune defenses against tuberculosis facilitated by DNA vaccines [45]. Moreover, the efficacy of a composite DNA vaccine containing six genes encoding key antigens from *M. tuberculosis* and *Brucella abortus* was evaluated, employing the DNA-IL-12 adjuvant system. Mice immunized with the DNA vaccine along with DNA-IL-12 exhibited significantly decreased bacterial burdens in the lungs and spleen upon challenge compared to those receiving the DNA vaccine alone [46]. The combined group demonstrated heightened antigen-specific immune responses, characterized by increased levels of IFN-γ, enhanced CD4^+^ and CD8^+^ T-cell responses, elevated IgG titers, and a Th1-skewed immune profile. These findings highlight the potential of IL-12 as an adjuvant in enhancing protective immunity against both *M. tuberculosis* and *B. abortus* [46]. 

In response to *M. tuberculosis* infection, the upregulation of IL-12, a pivotal factor in fostering Th1-type responses, drives the development of IFN-γ-producing T cells [112]. Utilizing IL-12 as an adjuvant in DNA vaccines targeting multiple pathogens demonstrated promising outcomes. While BCG can initiate Th1-type immune responses, the strength of this response has waned over time [43]. Integrating IL-12 as an immune adjuvant with BCG vaccination has significantly enhanced its protective efficacy against *M. tuberculosis* infection. IL-12 holds promise in enhancing the efficacy of BCG vaccination by bolstering the intensity of the Th1-type response prior to facing infectious challenges.

## 7. IL-21

IL-21, belonging to the IL-2 superfamily [113], binds to receptors on the surface of various immune cells such as T cells, B cells, NK cells, DCs, and keratinocytes, indicating a broad spectrum of biological effects [114]. IL-21 exhibits pleiotropic effects, ranging from enhancing T-cell proliferation and promoting the differentiation of B cells into memory cells and terminally differentiated plasma cells to boosting the function of natural-killer cells [113,114]. Intrinsic IL-21 signaling in CD4^+^ T cells is crucial for generating memory CD4^+^ T cells in vivo [115]. IL-21 has been demonstrated to synergistically interact with IL-10 in facilitating the development of memory CD8^+^ T cells [116]. The delineated biological effects of IL-21 on NK cells, CD8^+^ T cells, and B cells, in conjunction with its robust antiviral efficacy demonstrated in murine models, position it as a promising candidate for incorporation as a vaccine adjuvant [117].

Vaccination with a DNA vaccine pRSC-IL21-Ag85A (a plasmid co-expressing IL-21 and Ag85A) in mice demonstrated enhanced immune responses compared to those vaccinated with pRSC-Ag85A alone, performing the same level of efficacy of the BCG vaccination. This heightened response was comparable to the efficacy of BCG, indicating that IL-21 serves as a promising adjuvant to enhance the immunogenicity of tuberculosis DNA vaccines [47]. Furthermore, the same group developed another DNA vaccine containing a fusion protein of Ag85A, ESAT-6, and IL-21 (Ag85A-ESAT-6-IL-21) to assess its protective efficacy against *M. tuberculosis* in mice. Following intranasal DNA vaccine priming and BCG boosting, this strategy significantly increased NK cell and splenocyte cytotoxicity, elevated IFN-γ levels in the splenocyte supernatant, and enhanced sIgA levels in bronchoalveolar lavage compared to a DNA vaccine or BCG immunization alone. The heterologous prime-boost approach notably reduced bacterial loads in mouse lungs, highlighting a promising mucosal-targeted vaccination strategy against tuberculosis [48]. In addition, the cationic nanoparticle-based DNA vaccine Ag85A-ESAT-6-IL-21 exhibited a statistically significant enhancement in protective efficacy against *M. tuberculosis* infection compared to the DNA vaccine Ag85A-ESAT-6-IL-21 administered alone [49].

IL-21 can be generated by Th1-type and Th2-type cells and follicular CD4^+^ T cells, whose production is partially modulated by the specific microenvironment. The physiological effects of IL-21 are extensive, encompassing established impacts on B cells, CD8^+^ T cells, NK cells, and DCs [114]. The incorporation of IL-21 as an adjuvant in DNA vaccines has demonstrated substantial promise in augmenting immune responses against *M. tuberculosis*, underscoring the potential of IL-21 in enhancing the immunogenicity of tuberculosis DNA vaccines. Importantly, IL-21 exhibits a synergistic impact on the clonal expansion of CD8^+^ T cells when co-administered with either IL-7 or IL-15 [118]. Therefore, the selective combination of members within the IL-2 superfamily is promising for enhancing the investigation of tuberculosis vaccine adjuvants.

## 8. GM-CSF

The CSF family predominantly comprises three canonical members: macrophage (M)-CSF (or CSF-1), granulocyte (G)-CSF (or CSF-3), and GM-CSF (or CSF-2) [119]. GM-CSF exhibits various biological effects, with its key impacts in vaccination being the enhancement of maturation, migration, and immunostimulatory functions of Langerhans cells, dendritic cells, and NK cells [120,121,122]. Additionally, GM-CSF boosts MHC class II expression on APCs, which is crucial for the antigen presentation to CD4^+^ T-helper cells. GM-CSF increases the expression of CD80, a costimulatory molecule essential for T-lymphocyte activation, on Langerhans giant cells in vitro [123,124]. Furthermore, GM-CSF triggers a local inflammation at the injection site, leading to the recruitment of APCs [125]. GM-CSF has been employed as an adjuvant in vaccines to enhance immune responses against HIV and COVID-19 infections and in cancer vaccine formulations [126].

Researchers aimed to boost the immunogenicity of a plasmid DNA vaccine for tuberculosis by incorporating Ag85A and GM-CSF genes and employing electroporation as a delivery technique. The investigation revealed that electroporation facilitated comparable efficacy between a single intramuscular DNA injection and repeated injections in activating specific T cells. Concurrent expression of GM-CSF amplified T-cell activation and cytotoxic T-lymphocyte (CTL) activities. While electroporation alone conferred robust immune protection, GM-CSF expression moderately bolstered the systemic defense [50]. Additionally, in a murine model utilizing BCG priming and DNA vaccine boosting, the DNA vaccine expressing Ag85A and GM-CSF demonstrated a notable enhancement in cytotoxic T-lymphocyte activity, IFN-γ levels, and antibody titers compared to mice receiving BCG or standalone DNA vaccines. The BCG priming, sequentially followed by DNA vaccine boosting, provided adequate immune protection against the *M. tuberculosis* challenge [51]. The BCG, including AdGM-CSF (an adenoviral GM-CSF transgene-based adjuvant formulation), significantly amplified the potency and persistence of anti-mycobacterial Th1-type immunity compared to BCG alone or with a control vector. This improved vaccine formulation elicited a significant augmentation in mycobacterial antigen-specific IFN-γ releasing CD4^+^ T cells, enhancing immune protection against subsequent mycobacterial challenges [52]. Furthermore, researchers explored how the BCG vaccine strain that delivered GM-CSF (BCG:GM-CSF) influenced immunity against *M. tuberculosis*. BCG:GM-CSF boosted the production and activity of APCs derived from murine bone marrow, resulting in elevated levels of specialized immune cells and enhanced defense against *M. tuberculosis* infection [53]. Subsequently, the same group delivered BCG:GM-CSF to the lungs and noted an increase in pulmonary DC numbers and heightened secretion of IL-12, surpassing the effects of standard BCG immunization. This targeted strategy facilitated the rapid priming of antigen-specific CD4^+^ T cells in lymph nodes and promoted the migration of activated CD4^+^ T cells to the lungs [54].

The significant role of GM-CSF in modulating immune responses through its impact on the antigen presentation process has been well-documented in disease models encompassing both Th1-type and Th2-type immunities. Crucially, these investigations illustrate that GM-CSF does not alter the fundamental nature of immune responses from Th1-type to Th2-type or vice versa; instead, it enhances the immune response of either phenotype [127,128,129]. GM-CSF plays a vital role in recruiting lymphocytes, fostering a Th1-type response within the lungs, aiding in the formation of characteristic mononuclear granulomas, and notably contributing to the control of *M. tuberculosis* bacterial growth [130]. Supplementation of AdGM-CSF to BCG enhanced Th1-type immunity, bolstering defense against mycobacterial challenges. Furthermore, BCG:GM-CSF upregulated APCs production and activity, fortifying protection against *M. tuberculosis*. Localized administration of BCG:GM-CSF to the lungs augmented immune cell populations and IL-12 secretion, facilitating an effective immune response against *M. tuberculosis*. In conclusion, integrating GM-CSF with DNA vaccines targeting *M. tuberculosis*, particularly in conjunction with BCG priming, has shown significant promise in enhancing immune responses and protective efficacy, suggesting a strategic avenue for tuberculosis management.

**Figure 1:**
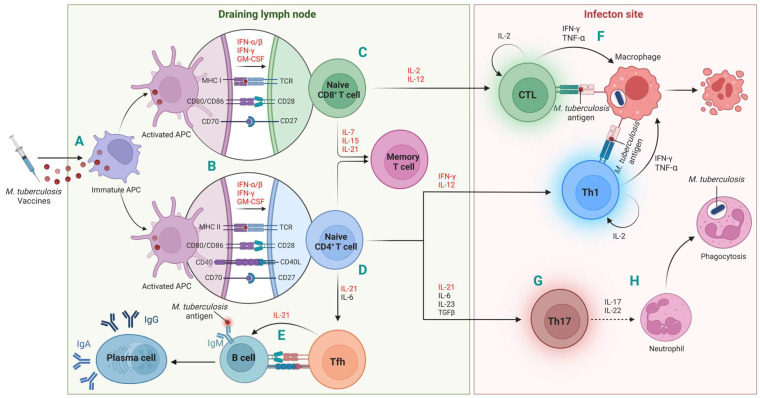
The potential roles of cytokines in potentiating immune responses induced by tuberculosis vaccines. (**A**) Following vaccination, *M. tuberculosis* (MTB) vaccine molecules containing cytokines are taken up by macrophages or dendritic cells (DCs). Antigen-loaded DCs are the primary antigen-presenting cells (APCs). APCs, together with free vaccine molecules and cytokines, migrate through the lymphatics to the draining lymph nodes (DLNs), initiating an anti-TB immune response; (**B**) Within the DLNs, mature APCs present MTB-antigen peptides on MHC class I and II molecules to naive CD8^+^ and CD4^+^ T cells, respectively. APC-provided costimulatory signals facilitate the induction of TB antigen-specific T-cell responses, such as those mediated by interactions between CD80/CD86–CD28, CD70–CD27, and CD40–CD40L. These co-stimulations can be augmented by potential adjuvants IFN-α/β, IFN-γ, and GM-CSF, respectively; (**C**) Presentation of antigens to CD8^+^ T cells by APCs leads to the differentiation of cytotoxic T-lymphocytes (CTLs), a process further bolstered by IL2 and IL-12, acting as an adjuvant; (**D**) Antigen presentation to CD4^+^ T cells by APCs results in the differentiation of various inflammatory T-cell subsets, including Th1 (facilitated by IFN-γ and IL-12), Th17 (promoted by IL-6, IL-21, IL-23, and TGF-β), and T follicular helper (Tfh) cells (assisted by IL-6 and IL-21). IL-15 and IL-7 play critical roles in preventing T cells from apoptosis during the T-cell activation stage, enabling the generation of memory T cells. IL-21 synergizes with IL-7 or IL-15 to promote the proliferation and survival of memory T cells; (**E**) B cells, activated either directly by TB vaccine antigens or with the assistance of Tfh cells (facilitated by IL-21), differentiate into plasma cells and secret IgG and IgA; (**F**) CTLs and Th1 cells migrate to the infection site, where they eliminate *M. tuberculosis*-infected macrophages through cytotoxicity and the secretion of effector cytokines like IFN-γ and TNF-α. Additionally, they secrete IL-2 to facilitate extensive self-amplification of T cells; (**G**) Th17 cells also migrate to the infection site, producing IL-17 and IL-22, which stimulate the production of neutrophil-attracting chemokines by respiratory epithelial cells (not illustrated); (**H**) Neutrophils engage in phagocytosis to eradicate the extracellular free *M. tuberculosis*. The cytokines highlighted in red are the focus of discussion in this review. This figure was created with BioRender.com.

In summary, Type I and Type II IFNs, interleukins such as IL-2, IL-7, IL-15, IL-12, and IL-21, along with GM-CSF used as an adjuvant, have the potential to significantly enhance the efficacy of tuberculosis vaccines by modulating various stages of the immune response, thereby augmenting their protective effects against tuberculosis (Figure 1).

## 9. Conclusions and Perspectives

The future of tuberculosis vaccinations may require strategically incorporating cytokines as adjuvants to optimize immune responses and bolster protection against *M. tuberculosis*. Cytokines, such as Type I IFNs, Type II IFN, IL-2, IL-7, IL-15, IL-12, IL-21, and GM-CSF, play crucial roles in regulating immune responses and have been investigated as potential adjuvants in tuberculosis vaccines. These cytokines have shown promise in enhancing immune responses, bolstering protective efficacy, and contributing to the development of enduring immunity against *M. tuberculosis*. Notably, their integration into vaccine formulations has demonstrated significant potential in augmenting vaccine-induced immune responses and protection against tuberculosis. Further research and clinical trials are warranted to elucidate the optimal dosages, formulations, and delivery methods of cytokine-adjuvanted vaccines. Additionally, exploring combinatorial approaches that harness the synergistic effects of multiple cytokines may further enhance vaccine-induced immunity. Recent advancements in vaccinology have introduced innovative technologies poised to transform vaccine development. mRNA vaccine technology has emerged as a powerful platform with the potential to revolutionize vaccine development due to its unique advantages. Incorporating cytokines into RNA vaccine formulations holds promise for directing the immune system to induce enhanced, enduring, and T/B-cell-balanced vaccine immunity. Defining the cytokine profile of RNA vaccines could enable customized immune responses tailored to the pathogen’s characteristics. Strategic cytokine utilization may also reduce the effective RNA dose for protective immunity, thereby mitigating associated adverse effects from higher doses. While leveraging cytokines as adjuvants in RNA vaccine prototypes presents substantial potential for refining vaccine efficacy and precision, it necessitates a comprehensive evaluation of biological effects, safety profiles, and regulatory compliance. Integrating mRNA vaccine technology with cytokine adjuvants in tuberculosis vaccine development offers a promising strategy to enhance immune responses, improve vaccine efficacy, and address the complex challenges associated with tuberculosis control. By leveraging the synergistic effects of mRNA vaccines and cytokine adjuvants, researchers can potentially develop more effective tuberculosis vaccines with long-lasting memory immunity that contribute to the global efforts to combat tuberculosis as a major public health concern. Lipid nanoparticles (LNPs) are indispensable delivery vehicles for mRNA vaccines. Nanoparticle vaccines, composed of natural or synthetic components, are capable of delivering multivalent antigens simultaneously while safeguarding stimulatory elements like cytokines at the periphery. This design allows for controlled release at target sites, alleviating adjuvant toxicity and ensuring vaccine efficacy. Overall, the exploration of appropriate cytokines as innovative adjuvants for the development of secure and powerful *M. tuberculosis* vaccines, combined with the utilization of novel antigenic candidates and advanced technologies, opens a promising avenue for tuberculosis prevention and treatment.

## Figures and Tables

**Table 1:** Cytokine-adjuvanted *M. tuberculosis* vaccines under preclinical studies.

Cytokines	Major Immunologic Functions	Cytokine-Adjuvanted Vaccines	Model	Mechanisms and Effects	References
Type I IFNs	Stimulates the maturation of APCs, elevates costimulatory signals, and augments their capacity for antigen presentation or cross-presentation	BCG + IFN-β	Cell culture	Enhancing Th1-type response and promoting DC maturation and IL-12-releasing	[26,27]
		BCG + IFN-α	Human	Boosting BCG-induced IFN-γ production in bladder cancer patients and enhancing BCG-induced IL-12 and TNF-α while reducing IL-10 levels	[28]
		BCG + IFN-α	Mouse	IFN-α in BCG-vaccine provided protection against *M. lepraemurium* infection in mice.	[29]
		BCG + IFN-α	Mouse	Enhancing specific Th1-type cytokine production in vitro and in vivo and leading to the reduction in bacterial burden after the *M. tuberculosis* challenge	[30]
Type II IFN	Stimulates APCs to enhance the expression of costimulatory molecules and cytokines essential for activating T cells	Six MTB antigens + Ribi + IFN-γ	Mouse	Elevating proliferation, IFN-γ secretion, and NO production in splenocytes, leading to a marked reduction in CFU counts upon exposure to *M. tuberculosis*	[31]
		BCG + Ag85B + ESAT-6 + IFN-γ	Mouse	Eliciting heightened specific antibody titers, bolstering cellular immune responses, and conferring comparable or superior protection against *M. tuberculosis* infection	[32]
		BCG + IFN-γ	Mouse	Improving bacterial clearance and diminishing tissue pathological changes at mycobacterial infection sites	[33]
IL-2	Promotes the proliferation and differentiation of effector T cells, memory T cells, and NK cells	BCG + ESAT-6 + IL-2	Mouse	Inducing robust Th1-type responses, marked by enhanced lymphoproliferation, IFN-γ secretion, and augmented cytotoxic T-lymphocyte functionality	[34]
		Hsp65 + IL-2	Mouse	Inducing robust antigen-specific immune responses, including IFN-γ release, and activation of CD4^+^ and CD8^+^ T cells, exhibiting superior protective and therapeutic effects	[35,36]
		BCG + IL-2	Mouse	Eliciting a Th1-type immune profile in both immunocompromised and IL-4 transgenic mice	[37]
IL-7 and IL-15	IL-7 is required for T-cell development and for maintaining and restoring homeostasis of mature T cells. IL-15 exhibits a wide array of functions in the modulation of both innate and adaptive immune responses, mirroring the activities of IL-2.	Flt3L-Mtb32 + IL-7-nFc	Mouse	Augmenting Mtb32-specific T-cell responses, decreasing *M. tuberculosis* reactivation following dexamethasone treatment, ameliorating lung pathology, and reducing pulmonary inflammation	[38]
		BCG + Ag85B + IL-15	Mouse	Elevating the levels of IFN-γ-producing CD8^+^ and CD4^+^ T cells, resulting in notable lung protection upon challenge with *M. tuberculosis*	[39]
		MVA + 5 MTB antigens + IL-15	Mouse	Exhibiting endure protective immunity lasting at least 16 months post-initial vaccination and demonstrating sustained protection on par with BCG immunization	[40]
		BCG + IL-7 + IL-15	Mouse	Amplifing the memory response of CD4^+^ and CD8^+^ T cells, elevating production of Th1-type cytokines, and significantly diminishing the mycobacterial load in the lungs	[41]
		LT70 + MH + IL-7-Linker-IL-15	Mouse	Augmenting the efficacy of tuberculosis subunit vaccines by strengthening central memory-like T cells	[42]
IL-12	Governs T-cell and natural-killer-cell responses, stimulates IFN-γ production, promotes the differentiation of Th1-type cells, and serves as a vital bridge between innate resistance and adaptive immunity	BCG + IL-12	Mouse	Slightly improving protection in the early stages and significantly enhancing protection in later stages	[43]
		BCG + IL-12	Mouse	Significantly decrease *M. tuberculosis* load via enhancing IFN-γ production in the spleen cells,	[44]
		Ag85B + IL-12	Mouse	Enhancing the protective efficacy against the *M. tuberculosis* challenge by amplifying T-cell responses	[45]
		Six MTB antigens + IL-12	Mouse	Reducing bacterial burdens in the lungs and spleen upon challenge, demonstrating heightened antigen-specific immune responses, characterized by increased levels of IFN-γ, enhanced CD4^+^ and CD8^+^ T-cell responses, and a Th1-skewed immune profile	[46]
IL-21	Enhances T-cell proliferation, promotes memoryand plasma B cell differentiation, and boosts the function of natural-killer cells	Ag85A + IL-21	Mouse	Enhancing immune responses, while performing same level efficacy of BCG vaccination	[47]
		Ag85A + ESAT-6 + IL-21	Mouse	Increasing NK cell and splenocyte cytotoxicity, elevating IFN-γ levels in the splenocyte supernatant, and raising sIgA levels in bronchoalveolar lavage	[48,49]
GM-CSF	Regulates growth and differentiation of hematopoietic cells, enhances the maturation, migration and immunostimulatory functions of Langerhans cells, dendritic cells, and NK cells	Ag85A + GM-CSF	Mouse	Moderately bolstering systemic defense by enhancing IFN-γ production from splenocytes	[50,51]
		BCG + GM-CSF	Mouse	Amplifiing the potency and persistence of anti-mycobacterial Th1-type immunity, augmenting antigen-specific IFN-γ-releasing CD4^+^ T cells, and enhancing immune protection against subsequent mycobacterial challenges	[52]
		BCG + GM-CSF	Mouse	Enhancing defense against *M. tuberculosis* infection by increasing pulmonary DCs and antigen-specific immune cells and heightening secretion of IL-12 upon pulmonary administration	[53,54]

## Data Availability

No new data were created or analyzed in this study. Data sharing is not applicable to this article.
